# Mechanisms of Slowed Aging in a New Model System for Extended Longevity

**DOI:** 10.1093/geroni/igaf122.531

**Published:** 2025-12-31

**Authors:** Jessica Foley, Stephen Montgomery

**Affiliations:** Tufts University, Medford, Massachusetts, United States; University of Bristol, Bristol, England, United Kingdom

## Abstract

Evolution has given rise to lifespans in extant species ranging from days to centuries. Given that mechanisms of aging are highly conserved, studies of long-lived lineages across the animal kingdom could shed light on mechanisms of healthy aging in humans. However, typical animal models of extended lifespan often live for decades, making them intractable for longitudinal studies. Ideal model systems would instead be organisms that are long-lived relative to their close evolutionary relatives, yet have lifespans on experimentally tractable scales. Lifespans in the Heliconius butterfly genus, reaching nearly a year, are among the longest recorded in butterflies. This represents a dramatic extension over their closest relatives in the Heliconiini tribe, which typically live only 1–2 months. While previous work has attributed this difference to a plastic response to enhanced nutrition, the degree and consistency of the extension across the genus led us to hypothesise that it reflects evolved, heritable mechanisms of longevity. We conducted detailed survival and functional senescence analyses on two representative shorter- and longer-lived species to show evidence for evolved mechanisms for slowed ageing in Heliconius. Results of transcriptomic analyses then revealed a strong upregulation of pathways related to protein-folding in aged Heliconius, suggesting that an increased investment into protoeostatic mechanisms may have been a key factor in their evolution of longevity. This finding aligns with extensive evidence that proteostasis plays a central role in aging, including in humans, and highlights the value of studying diverse organisms to advance understanding of healthy aging.

